# A critical review and development of a conceptual model of exclusion from social relations for older people

**DOI:** 10.1007/s10433-019-00506-0

**Published:** 2019-02-20

**Authors:** Vanessa Burholt, Bethan Winter, Marja Aartsen, Costas Constantinou, Lena Dahlberg, Villar Feliciano, Jenny De Jong Gierveld, Sofie Van Regenmortel, Charles Waldegrave, The Working Group on Exclusion from Social Relations, part of the COST-financed Research Network ‘Reducing Old-Age Exclusion: Collaborations in Research and Policy’ (ROSENet)

**Affiliations:** 1 Centre for Innovative Ageing, Swansea University, Swansea, Wales UK; 2 Centre for Welfare and Labour Research, OsloMet Oslo Metropolitan University, Oslo, Norway; 3 University of Nicosia Medical School, Nicosia, Cyprus; 4 School of Education, Health and Social Studies, Dalarna University, Falun, Sweden; 5 Aging Research Center, Karolinska Institute, Solna, Sweden; 6 Department of Development and Educational Psychology, University of Barcelona, Barcelona, Spain; 7 Faculty of Social Science, VU University Amsterdam, Amsterdam, The Netherlands; 8 Netherlands Interdisciplinary Demographic Institute, The Hague, The Netherlands; 9 Faculty of Psychology and Educational Sciences, Vrije Universiteit Brussel, Brussels, Belgium; 10 Family Centre Social Policy Research Unit, Lower Hutt, New Zealand

**Keywords:** Disadvantage, Later life, Knowledge synthesis, Old-age exclusion, Social relations

## Abstract

Social exclusion is complex and dynamic, and it leads to the non-realization of social, economic, political or cultural rights or participation within a society. This critical review takes stock of the literature on exclusion of social relations. Social relations are defined as comprising social resources, social connections and social networks. An evidence review group undertook a critical review which integrates, interprets and synthesizes information across studies to develop a conceptual model of exclusion from social relations. The resulting model is a subjective interpretation of the literature and is intended to be the starting point for further evaluations. The conceptual model identifies individual risks for exclusion from social relations (personal attributes, biological and neurological risk, retirement, socio-economic status, exclusion from material resources and migration). It incorporates the evaluation of social relations, and the influence of psychosocial resources and socio-emotional processes, sociocultural, social-structural, environmental and policy contextual influences on exclusion from social relations. It includes distal outcomes of exclusion from social relations, that is, individual well-being, health and functioning, social opportunities and social cohesion. The dynamic relationships between elements of the model are also reported. We conclude that the model provides a subjective interpretation of the data and an excellent starting point for further phases of conceptual development and systematic evaluation(s). Future research needs to consider the use of sophisticated analytical tools and an interdisciplinary approach in order to understand the underlying biological and ecopsychosocial associations that contribute to individual and dynamic differences in the experience of exclusion from social relations.

## Introduction

Social exclusion of older people has been defined as ‘a complex process that involves the lack or denial of resources, rights, goods and services as people age, and the inability to participate in the normal relationships and activities, available to the majority of people across the varied and multiple domains of society. It affects both the quality of life of older individuals and the equity and cohesion of an ageing society as a whole’ (Walsh et al. 2017). There is a growing body of literature on social exclusion in later life which could be taken as an indication of the value that the concept has as an explanatory framework for understanding disadvantage in later life.

Several domains that contribute to old-age social exclusion have been identified. These include exclusion from material and financial resources, social relations, services, amenities and mobility, civic participation, neighbourhood and community and sociocultural aspects of society (Walsh et al. 2017). There have been some attempts to look at the conceptual relationships between drivers and outcomes of social exclusion and the interrelationship between the domains of exclusion (e.g. Barnes et al. 2006; Scharf et al. 2005; Walsh et al. 2012). However, there has been relatively little attention paid to a similarly detailed conceptualization of each domain.

The recent proliferation of policy-addressing social isolation of older people (e.g. Department for Digital Culture Media and Sport 2018; Ministry of Social Development 2001; World Health Organization 2007) highlights the importance of developing a robust conceptualization of this domain of social exclusion. While the volume of the body of evidence on exclusion from social relations ranked third in a recent review (Walsh et al. 2017), the capacity to develop evidence-based policy and/or practice interventions is hampered by a lack of understanding of the relationship between elements that comprise this domain.

Responding to this gap in knowledge, the aim of this paper is to undertake a critical review to take stock of the literature on exclusion of social relations for older people. In this critical review, the literature is interpreted by experts in order to identify the risks for, influences on, and the outcomes of exclusion from social relations and to propose a conceptual model to explain the relationships between these factors. A critical review does not attempt to aggregate all of the evidence on a subject. Instead, in this review, we use selected examples of the literature to demonstrate themes that serve as conceptual contributions to an overall model.

**Fig. 1:**
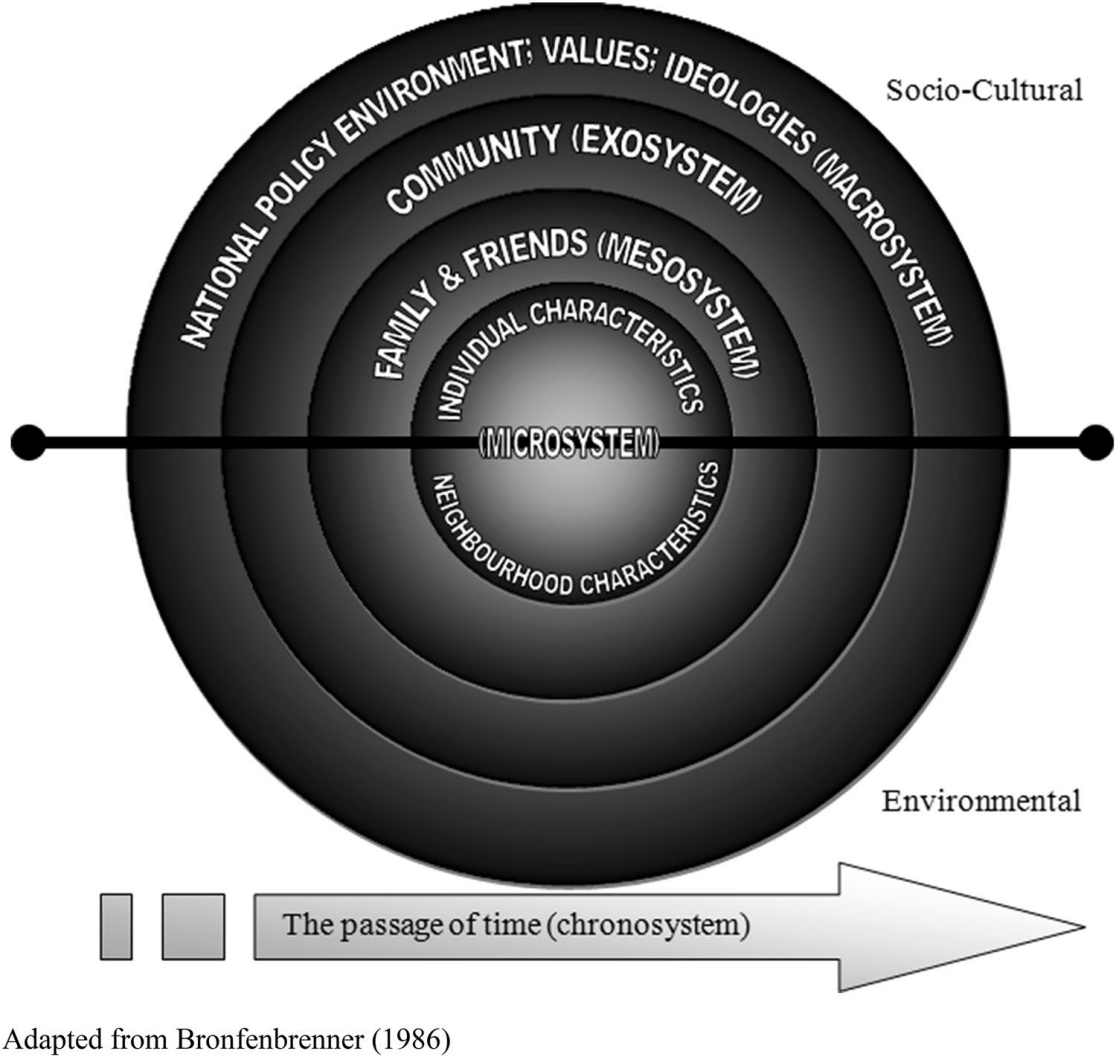
Human ecological framework. Adapted from Bronfenbrenner (1986)

We define social relations as comprising social resources, social connections and social networks. More formal types of engagement with social organizations are not included in this definition, as they are conceptually incorporated in the domain of cultural and civic participation. In this article, we frame exclusion from social relations within a critical human ecological framework. From the critical human ecology perspective, the biological manifestation of the body, psychological traits and the sociocultural, social-structural, policy and physical environment fundamentally impact on the human experience, whilst simultaneously individuals shape or adapt their environments in both the physical and sociocultural milieux in which they are situated (Bronfenbrenner 1986). The human ecology framework guides the synthesis of evidence and takes into account the interrelatedness of the macro-, exo-, meso-, micro- and chronosystems to transform our understanding of exclusion from social relations (Fig. 1).

In synthesizing the literature within the human ecological framework, we address the questions:

1. What are the risks for exclusion from social relations for older people?
2. What factors (other than risks) impact on exclusion from social relations for older people?
3. What are the outcomes of exclusion from social relations for older people?
4. How can the evidence be organized to develop a conceptual model of exclusion from social relations?

## Methods

### Critical review

Drawing on review processes well suited to theory generation, a critical review of the evidence was conducted (Grant and Booth 2009). A critical review provides insight into the dynamics underlying the findings of individual studies that often only capture single relationships (e.g. between a risk factor and social isolation). A critical review simultaneously considers the complexity of the issue and other possibilities to form theory and integrates, interprets and synthesizes information across studies (Finfgeld-Connett 2014). The critical review is not as systematic as other forms of review. For example, there is neither an expectation that search terms and search methods (e.g. databases searched) are presented nor a formal assessment of the quality of studies. This is because the emphasis is on the contribution of the material to the development of a conceptual model. Model development relies heavily on the experience and expertise of the reviewers. The resulting model is a subjective interpretation of the literature and is intended to be the starting point for further evaluations, that is, ‘a “launch pad” for a new phase of conceptual development and subsequent “testing”’ (Grant and Booth 2009).

In this critical review, a narrative format was used to summarize the evidence concerning exclusion from social relations. Interpretations of the interconnections (including linear, circuitous, hierarchical and temporal relationships) between domains were explicated in diagrammatic form (Grant and Booth 2009).

#### Step 1: Developing the initial conceptual model

In October 2016, an expert review group (ERG) met at the National University of Ireland, Galway, and reviewed the evidence concerning exclusion from social relations that were identified in a draft of the scoping paper published by Walsh et al. (2017). Walsh et al. searched gerontological literature comprising peer-reviewed journal articles, books and research reports published between 1997 and 2016 that focused on older people (aged 50 years and over). Stage one of the review included documents concerned with conceptual frameworks of old-age exclusion, and stage two included documents that referred to particular domains of exclusion.

The evidence from Stage 2 of the scoping review that related to exclusion from social relations (114 articles) was organized by the ERG, into a draft conceptual model that disentangled risks for exclusion from distal outcomes of exclusion, and the contexts that may impact on the process of exclusion. The effects of time or life-course dynamics on exclusion from social relations were added to the model. The draft conceptual model took into account interrelationship between systems in the critical human ecology framework (Fig. 1). A diagram of the draft conceptual model was produced and discussed within the ERG. This approach to theory generation and model development is common when reviewers have extensive knowledge of a topic, and/or where they have undertaken primary research on the topic (Hsieh and Shannon 2005). Using expert opinion, gaps in the original scoping review were identified.

#### Step 2: Additional review and model refinement

In Step 2, a subset of the ERG (the authors, with expertise in social relations, social exclusion, loneliness, environmental gerontology, cultural exclusion, cognitive function, biomedicine, health care, social work, social care, poverty, social policy, generativity, volunteering and sexual expression) reviewed additional literature relating to the conceptual model. Search terms were used to iteratively identify articles for inclusion in the review (quantitative and qualitative studies that were published or ‘grey’ literature) that addressed areas of the conceptual model that were absent from the original scoping review by Walsh et al. (2017). Search keywords were derived from the established literature on exclusion from social relations and included: social exclusion; social relations; intergenerational relations; social networks. Keywords relating to ageing and older people included: ag(e)ing; older persons; older adults; seniors; elderly; elders; senior citizens. Keywords specific to domains in the conceptual model were also generated (e.g. retirement, resilience, social cohesion). The ERG considered the weight of the evidence in order to explain how domains of the conceptual model fitted together but also to identify inconsistencies and gaps. Based on a process of constant iterative reflection that involved sending drafts of the article and the conceptual model between authors for revision, formative ideas and interconnections were examined through multiple lenses. Consequently, the review resulted in amendments to the draft model as it was tested and interpreted with the review evidence (Finfgeld 2003).

#### Step 3: Endorsement of the final conceptual model

The revised conceptual model was presented to the full ERG at Masaryk University, Brno, Czech Republic in September 2017, and discussion and feedback were invited. This process helped ensure potentially significant elements of the conceptual model were not omitted. Thus, the iterative process of expert input and model refinement continued until the ERG felt that the synthesized review findings resonated with the final conceptual model and their interpretations of the data. This article represents a synthesis of the findings that has been endorsed the by ERG comprising 45 members from 25 countries.

## Results

**Fig. 2:**
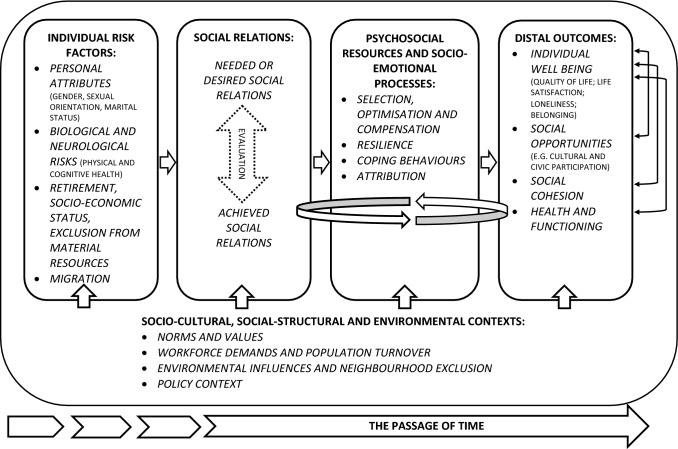
Conceptual model of exclusion from social relations for older people

The final conceptual model of exclusion from social relations for older people as developed through the critical review and interpretation of the data by the ERG is outlined in Fig. 2. In line with the elements of the conceptual model, we start by describing the current knowledge on risks to exclusion from social relations. Next, we report on the connection between objective ratings and subjective assessments of social relations. This is followed by a summary of the role of psychological resources and socio-emotional processes in mediating or moderating exclusion from social relations. The evidence of sociocultural, social-structural, environmental and policy contextual influences on exclusion from social relations is summarized. We report on the evidence concerning the outcomes of exclusion from social relations whereby individual well-being (e.g. quality of life, life satisfaction, loneliness and belonging); health and functioning; social opportunities and social cohesion are conceptualized as distal outcomes. Finally, we report on the dynamic relationship between elements of the model.

Each section of the results starts with a summary of the ERG’s interpretation of the literature and how this is situated in the ecological framework (Fig. 1). The summary is followed by illustrative examples of the published studies that were used to inform the interpretation.

### Individual risk factors

Studies consistently reported that certain individual characteristics or life events impact on exclusion from social relations for older people. These risks include personal attributes (gender, sexual orientation and partner status); biological and neurological factors; retirement, socio-economic status and exclusion from material resources; and migration.

#### Personal attributes: gender, sexual orientation and partner status

Personal attributes such as gender, sexual orientation and partner status are part of the microsystem in the human ecological framework (Fig. 1). Some research has demonstrated that older women have more kin in the social circles, but that there is no difference between older men and women in the non-kin members of their networks (McPherson et al. 2006). While social isolation is more common for older women than men (Wenger et al. 1996), the differences are largely due to differences in marital status with women more likely to be widowed and living alone.

Being married offers a greater degree of protection against exclusion from social relations for older people than other marital states (De Jong Gierveld et al. 2009). While spousal bereavement results in termination of a key relationship that usually provides an ‘exclusive, close and intimate tie’ (Dykstra and Fokkema 2007), divorce and relationship breakdown can also have a negative impact on social interaction (Wenger 1996). On the other hand, some research indicated that widowhood may precipitate gains in social relations, with relatives providing greater social support following the death of a spouse (Ha 2008). On the whole, the weight of evidence suggests that widowhood and divorce are risk factors for exclusion from social relations for older people (Arber 2004).

Sexual orientation can also impact on exclusion from social relations for older people (Cronin and King 2010). However, the research tended to emphasize intersectionality of sexual orientation, place and discrimination which is addressed below.

#### Biological and neurological risks: physical and cognitive health

The ERG considered poor physical and cognitive health as biological or neurological risks for exclusion from social relations. However, health and functioning are also potential outcomes of exclusion from social relations (see below).

Negative life events such as poor health, impairment or pain impact on the ability to maintain usual lifestyles including customary levels of social interaction for older people (Bertoni et al. 2015; Coyle et al. 2017; Croda 2015; Hilaria and Northcott 2017). Furthermore, biological or genetic factors may impact on fertility and contribute to involuntary childlessness and fewer social relations (Lechner et al. 2007).

The effect of involuntary childlessness, social relations and psychological distress has been studied in younger populations. However, the distinction between involuntary and voluntary childlessness (that is, exclusion versus choice) has rarely been addressed in research with older populations (e.g. Klaus and Schnettler 2016). There are some notable exceptions, for example, Gibney et al. (2017) used childhood health status as a proxy for involuntary childlessness, and a special issue of the Journal of Family Issues addressed all types of childlessness in older age (Dykstra and Hagestad 2007). In general, in European studies, it has been assumed that childlessness in later life is involuntary, attributable to infertility or disruptions to marital status such as war (Gibney et al. 2017). Childlessness, as a deviation from cultural normative family forms, is reported below.

#### Retirement, socio-economic status and exclusion from material resources

Socio-economic status and exclusion from material resources are individual attributes that are part of the sociocultural microsystem in the ecological framework. However, they can also be considered as outcomes of interactions between macrolevel social policies (e.g. retirement) and environmental policies (e.g. rural/urban agricultural or industrial policies) that evolve over time (chronosystem) (Fig. 1).

Research has indicated that older people with higher education have more non-kin in networks and fewer kin than those with lower educational levels (McPherson et al. 2006). Other authors found a correlation between socio-economic status and loss of networks members: older people with a low socio-economic status were more likely to lose social relations through life events such as death and relationship breakdown, and less likely to match these losses with replacements than older people with a higher status (Cornwell 2015). Hietanen et al. (2016) suggest that this may be because childhood socio-economic status shapes social engagement in later life. However, across a range of countries, material deprivation and poverty limit full participation in the social life of communities for older people, limiting opportunities to optimize and diversify social interaction, and contributes significantly to exclusion from social relations (Ajrouch et al. 2005; Ellwardt et al. 2014; Fokkema et al. 2012; Stephens et al. 2010).

Many older adults living in Europe enter retirement with more resources than previous generations. However, retirement precedes reduced economic productivity (Moffatt and Heaven 2016) and can contribute to a decline in material resources which indirectly influence social relations. Retirement also has a direct impact on social interaction, which may be gendered. Research has demonstrated that following retirement, older men experienced a decline in social relations, while women increased social relations (Patulny 2009).

Following retirement, it is important for retirees to develop meaningful social roles as these can have a potential positive effect on well-being (Heaven et al. 2013). The difference in social relations observed between male and female retirees (e.g. Patulny 2009) may be explained by the alternative social roles on which they can successfully draw. Men are more likely to solely identify with a paid role in employment that is abrogated on retirement, whereas women often have alternative identities to draw upon including caring and unpaid voluntary and community work (Duberley et al. 2014). Gilleard and Higgs (2000) suggest that active construction of a ‘postwork’ identity may help to counter the high likelihood of exclusion from social relations for retirees whose identities are closely bound to their work roles.

#### Migration

Individual characteristics in the microsystem (e.g. resources, personality) of the human ecological framework interact with the chronosystem (encompassing environmental, social, cultural and political events occurring previous to, and throughout he life time of a person) to influence migration (Fig. 1). Migration itself is a risk for exclusion from social relations for older people.

The review indicated that migration within or across national boundaries can impact upon and disrupt social and support networks comprising kin and non-kin (Burholt 2004a; Burholt and Sardani 2017; De Jong Gierveld et al. 2015). While within country migrants may take time to establish local social relations after relocation (Walters and Bartlett 2009), for immigrants, developing new social networks in the country of settlement may be compromised by lack of language fluency (Wong et al. 2005).

Some research has indicated intersectionality between gender and fluency of language for older people from ethnic minority groups, create overlapping and interdependent systems of discrimination or disadvantage. In Europe, within immigrant groups there are gender differences in education and fluency in the national language of the host country (Burholt 2004b), which negatively affect women’s employment prospects. In turn, lower levels of employment (and language skills) impact on ethnic women’s social relations with others outside of their particular ethnic group. Thus, intersectionalities of socio-economic status, culture and gender can result in exclusion from social relations in later life (Viruell-Fuentes et al. 2012). However, this Western interpretation of exclusion from social relations may not be applicable across cultures. Maynard et al. (2008) have argued that older women from ethnic minority groups prioritize shared within-group identities, language and culture linked to kinship roles, rather than valuing integration into society. This is reported in more detail below, in relation to the influence of norms and values on exclusion from social relations for older people.

### Social relations

Social relations are part of the sociocultural microsystem in the ecological framework and are central to the conceptual model of exclusion from social relations for older people. Evidence suggests that *good* and *extensive* social relations with a range of people and groups including family, friends, neighbours and community groups foster social inclusion (Barnes et al. 2006). The ERG interpretation of the evidence suggested that judgements concerning both the quantity and quality of social relations are important, and in order to understand these, we need to consider both objective and subjective experiences.

Objective measurements of achieved social relations include the size of network, frequency of contact and physical distance between friends, neighbours and relatives (e.g. Lubben and Gironda 2000; Wenger 1991). There are more studies evaluating the impact of the quantity of social relations rather than the quality of relationships on outcomes for older people (Pinquart and Sorensen 2001). Studies evaluating the quality of relationships often consider how close a person feels to others (emotional distance) (e.g. Schwartz and Litwin 2018).

The negative impact of non-supportive, harmful or abusive relationships contributes to exclusion from social relations for older people. Elder abuse or mistreatment includes psychological, physical, sexual and financial abuse as well as neglect (Pillemer et al. 2016). Systematic reviews have shown that elder abuse is associated with fewer social relations and social isolation (e.g. Dong 2015). In turn, elder abuse is associated with adverse outcomes such as greater levels of loneliness (Dong et al. 2013), poor individual well-being (Dong 2015), health and functioning, such as more severe mental health issues, psychological distress, anxiety and suicidal thoughts (Yunus et al. 2017).

Central to the ERG’s understanding of exclusion from social relations for older people is that individual outcomes are dependent on personal assessments and judgements concerning the quality and quantity of relationships and whether these meet personal needs or desires (e.g. Shiovitz-Ezra 2015). Thus, one can experience exclusion from social relations (infrequent and poor quality social relations), but this only adversely impacts on outcomes if the experience deviates from personal expectations. Consequently, avoiding poor quality of life, lower levels of life satisfaction and loneliness may entail addressing the mismatch, by adjusting either *expectations* regarding the quality and frequency of social relations or *achieved* quality and frequency of social relations to balance both elements. Exclusion from social relations does not necessarily result in permanently poor outcomes across the life course. For example, some studies have shown the experience of loneliness after widowhood declines over time (Wenger et al. 1996), suggesting that older people adjust either their levels of social relations or expectations about types of relationships that are feasible or likely (Peplau and Caldwell 1978).

### Psychological resources and socio-emotional processes

Psychological resources and socio-emotional processes are subsumed in the microsystem but influence other levels of the ecological framework (Fig. 1). In later life, there is greater likelihood of experiencing disruptive losses, such as widowhood or a decline in material resources, that may contribute to exclusion from social relations and poor outcomes, if not managed. In the ERG’s conceptualization of exclusion from social relations for older people, psychological resources and socio-emotional processes can modulate the evaluation of achieved social relations (contrasted to needs and desires) and the experience of exclusion from social relations (Fig. 2). Psychological resources and socio-emotional processes influence how older people manage difficult situations and their preparedness or adaption to produce positive outcomes. The ERG’s conceptualization of the role of psychological resources and socio-emotional processes in social exclusion informed the subsequent review of the literature.

#### Selection, optimization and compensation

The process of selection, optimization and compensation (SOC) (Baltes and Carstensen 2003) has been associated with distal outcomes such as life satisfaction and quality of life (Freund and Baltes 1998). Selection refers to setting life goals that are salient to the individual. For example, studies have demonstrated that older people may terminate peripheral social relationships to focus on those that are particularly important (Lang 2000), which in terms of the conceptual model demonstrates simultaneous adjustment of achieved and desired social relations. Optimization and compensation refer to the processes of cognitive reappraisal that are adopted to achieve life goals (Urry and Gross 2010). For example, optimizing reduced social contacts may be achieved by maintaining reciprocal emotional support exchanges (Lang and Carstensen 1994), while a move into supported living environment may compensate for social losses (Burholt et al. 2013).

#### Resilience

In social psychology, resilience has been defined as ‘the process of effectively negotiating, adapting to, or managing significant sources of stress or trauma, […] and ‘bouncing back’ in the face of adversity’ (Windle 2011). Little is known about resilience in later life when faced with prolonged exposure to an adversity, such as exclusion from social relations. However, Marmot et al. (2012) have speculated that resilience moderates the way in which people experience social relationships and influence health outcomes.

#### Coping resources

Research has demonstrated that a range of coping strategies can be employed by older adults to moderate the experience of social relations, especially social isolation. The strategies employed include problem-focused strategies aimed at improving the quality and/or quantity of social relations; emotion-focused strategies aimed at lowering expectations for social relations or providing a distraction from social isolation, or meaning-focused coping strategies that draw on the individual’s beliefs and value systems (Peplau and Perlman 1982; Pettigrew and Roberts 2008; Rokach and Brock 1998; Schoenmakers et al. 2015). On the whole, research has focused on the difference between strategies in terms of the impact on loneliness and has found that employing a mix of coping strategies is most beneficial in dealing with the outcomes of exclusion from social relations and avoiding negative outcomes (Morgan 2015). The influence of coping strategies on modulating the interactions between personal characteristics, resources and life events along the path to other distal outcomes in later life has not been examined.

#### Attributions

In the ERG’s conceptual model (Fig. 2), attributions through social comparison may amplify poor outcomes stemming from exclusion from social relations for older people. Social comparison represents an interaction between the microsystem (individual characteristics), the mesosystem and/or macrosystem (Fig. 1). Social comparison theory (Festinger 1954) posits that social and personal worth are determined by perceptions of how others fare in relation to one’s own position. Comparisons can be made with someone who is perceived to be ‘better off’ (upward comparisons), ‘worse off’ (downward comparisons) or of the same status (lateral comparisons).

The traditional view of social comparison is that people deliberately or explicitly select and compare themselves to standards that are often similar to the self (Festinger 1954). However, more recent evidence suggests that the process is spontaneous and comparison is carried out automatically or implicitly (Mussweiler et al. 2004). Upward, downward or lateral comparison may not involve deliberate selection of a *similar* standard, but instead automatic selection can be representative of an extreme state. Consequently, dominant negative societal discourses about ageing, or diseases associated with old age, may be internalized and self-evaluations assimilated towards this stereotype (Burholt et al. 2016c). Neither automatic comparison (Mussweiler et al. 2004) nor unrealistic optimistic social comparison (Bortolotti and Antrobus 2015) is likely to yield useful information, and in the case of expectations, social relations may contribute to poor outcomes for older people. Additionally, cognitive processes (cognitive impairment, depression, anosognosia) may hamper optimal regulation through social comparison (Burholt and Scharf 2014; Burholt et al. 2016c).

### Sociocultural, social-structural and environmental contexts

The ERG reviewed and interpreted the evidence from the macrosystem which indicated that sociocultural, social-structural and environmental factors impact on the risks, social relations, psychological resources and socio-emotional processes and distal outcomes. Therefore, the interplay between norms and values (including prejudice, discrimination and ageism); workforce demands and population turnover; environmental influences and neighbourhood exclusion; and the policy context were incorporated into the conceptual model (Fig. 2).

#### Norms and values

Cultural norms and values refer to the typical or ‘normal’ set of beliefs, attitudes and patterns of behaviour that exist in a given group, community or society and are part of the sociocultural macrosystem in the ecological framework (Fig. 1). There is an emerging body of literature about older people, culture and social exclusion, focussing on such aspects as ageism, age discrimination, negative representations and social constructions of ageing, symbolic and discourse exclusion and identity exclusion (Walsh et al. 2017; Winter and Burholt 2018).

The subjective evaluation of social relations by older people is influenced by cultural values concerning the normative expectations for the ‘ideal’ levels and types of relationships. Norms can influence trends in marriage patterns and levels of fertility which in turn impact on the composition of families and the availability of support and emotional support from family members (De Jong Gierveld et al. 2018). As noted earlier, childlessness may also be considered a deviation from cultural norms as in Western and non-Western societies there are strong expectations concerning parenthood (Gibney et al. 2017). However, cultural values vary between societies. For example, the preferred configuration of networks of family and friends differs between individualist and collectivist cultures (Burholt and Dobbs 2014; Burholt et al. 2017). Research with older migrants from collectivist cultures suggests that family focused networks with few non-kin members represent the desired standard for social relations (Burholt et al. 2017; De Jong Gierveld et al. 2015). On the other hand, diverse networks comprising friends, family and incorporating community engagement are the preferred form in individualistic cultures (Fiori et al. 2006; Litwin and Shiovitz-Ezra 2010).

In addition to normative expectations concerning the configuration of social networks for older people, geographic locations are also subject to a set of normative expectations. For example, the rural idyll portrays rural life as bucolic and virtuous. However, mythologized normative expectations about rural living are, or are not achieved by subgroups with different modes of power relating to age, gender, marital status, health, class and in diverse rural settlement types (Burholt et al. 2018; Winter and Burholt 2018).

Influence of norms and values on institutionalized patterns of behaviour can lead to structural discrimination excluding groups or individuals from the same opportunities for social relations that are available to the majority of the population. Ageism is manifest in stereotypes that portray older people as weak, disabled or sick and contributes to widespread discrimination against older people influencing access to social relations (Palmore 2015). Some older people internalize negative age stereotypes. This has a deleterious impact on ‘self-perception’ which in turn has an adverse impact on social relations for older people (Vitman et al. 2014). Similarly, experiences of racial discrimination (Burholt et al. 2016a; Viruell-Fuentes et al. 2012), homophobia and heterosexism (Butler 2017; Villar et al. 2014) and the stigmatization of certain disabilities [e.g. dementia (Burholt et al. 2016c)] impact on social participation, social relations, and distal outcomes.

#### Workforce demands and population turnover

Workforce demands (increased female participation in the labour force, or lack of local employment opportunities) and resulting migration and population change within a community can influence exclusion from social relations for older people (Burholt and Sardani 2017). While migration is a life event that impacts on individual risk of experiencing exclusion from social relations (see above), it is also a macrolevel phenomenon that takes place in the exosystem of the human ecological framework (Fig. 1).

There are spatial differences in migration, for example area deprivation influences the perceived desirability of an area, and thus population stability or turnover (Stockdale 2010). Consequently, there are differences in exclusion from social relations in terms of ‘ageing in place’ (older people in communities of origin experiencing the out-migration of younger people) and ‘ageing places’ (communities that have a growing population of older in-migrants) (Burholt and Sardani 2017). While geographic labour mobility may impact on population turnover, other demands of the workforce [longer working hours, extended working year (Van der Hulst 2003) and job insecurity (Sverke and Naswall 2006)] may also impact on availability of family and friends to spend quality time with older people. Similarly, kin and non-kin caregiving can impact on the time available to maintain friendships and social relations (Rozanova et al. 2012).

#### Environmental influences and neighbourhood exclusion

Exclusion from social relations for older people is influenced by the environment and neighbourhood exclusion and is a product of the interaction between the environmental macrosystem and the exosystem in the ecological framework (Fig. 1). Thus, place, as a socio-spatial phenomenon, can shape older adult’s lives and can amplify or protect from exclusion from social relations.

Physical environments (exosystem) have an important influence on exclusion from social relations for older people. For example, neighbourhood design, housing diversity, population density, mixed land use and open space are associated with walkability and social contact (Burholt et al. 2016b). In these instances, activity levels are moderated by an individual’s ability to cope with *environmental stress* or hazards. Crime and fear of crime may also reduce accessibility and increase social isolation for older people (Acierno et al. 2004; Portacolone et al. 2018) and are influenced by *neighbourhood disorders* such as litter, graffiti and lighting (Lorenc et al. 2012) or the presence of security and neighbourhood watch signs (Kim and Clarke 2014). *Environmental stress* and *neighbourhood disorders* may impede older people’s access to the immediate environment, subsequently interfering with efforts to maintain or develop social relations (Burholt et al. 2016b).

There are different degrees of marginalization in disadvantaged and rural and remote places that may have fewer facilities and services. These can negatively influence social participation and social engagement of older people (Burholt and Scharf 2014). While some authors have noted that exclusion from social relations is particularly pronounced for those living in deprived and remote rural areas (Walsh et al. 2012), Scharf et al. (2005) found that older people living in deprived urban areas are more vulnerable to exclusion from social relations than those living in the UK as a whole.

#### Policy context

Policies are part of the macrosystem in the sociocultural or environmental milieu in the human ecological framework (Fig. 1). The ERG’s interpretation of the literature was that policies that tackle the risk for exclusion or the cultures and geographical locations in which exclusion takes place (e.g. discrimination, environment and access to services) have the potential to exert a positive influence on social relations for older people.

Welfare expenditure, particularly on care and health services, has the potential to cancel out some negative effects of exclusion from social relations for older people (Ellwardt et al. 2014). For example, Ogg and Renaut (2012) suggest that policies supporting intergenerational solidarity [e.g. cash for care (Da Roit and Le Bihan 2010)] could help maintain inclusive social relations. Additionally, policies that promote advice on welfare rights can modestly increase material resources and positively influence social relations for older people (Moffatt and Scambler 2008). However, these initiatives require stable economies and in times of austerity and economic recession are unlikely to be achieved. Cutbacks in care and greater reliance on kin and non-kin care make older people at risk of exclusion more vulnerable. One study showed that older people that needed support but did not receive kin or non-kin care and were not provided with formal care experienced higher levels of exclusion than care receivers and people with no care needs (Dahlberg and McKee 2016).

Certain rural policies encourage communities to take responsibility for governance and tackling local problems [e.g. Rural White Paper for England (House of Commons 1996); Positive Rural Futures, in Queensland, Australia (Herbert-Cheshire 2000); and Quebec’s National Policy on Rurality (Affaires Municipales et Régions Québec 2006)]. However, rural communities and the inhabitants therein have varying abilities to live up to the ‘self-help’ stereotype (Woods and Goodwin 2003). Communities that are unable (or unwilling) to provide services and amenities from informal, community and voluntary sources are not capable of protecting against exclusion from social relations for older residents (Burholt et al. 2018).

### Distal outcomes

The distal outcomes are the emergent products of a system, in which macro-, exo-, meso- and microsystems of the human ecological framework are inextricably connected to objective and subjective experiences of exclusion from social relations (Fig. 1). The ERG found that the most commonly cited outcome of exclusion from social relations for older people is loneliness (Victor et al. 2005). However, there is evidence that other outcomes that may be equally as important to the individual or society but are less well documented than loneliness. Along with other forms of individual well-being (quality of life, life satisfaction and belonging), social opportunities, social cohesion and health and functioning were incorporated into the conceptual model (Fig. 2).

#### Individual well-being: quality of life, life satisfaction, loneliness, belonging

Personal experiences of well-being are the heart of the ecological model (Barton and Grant 2006). Individual well-being such as quality of life, life satisfaction, loneliness and belonging are part of the microsystem in the ecological framework (Fig. 1).

Good social relations contribute to well-being and a good quality of life for older people (e.g. Gallagher 2012). Contact with relatives, neighbours and friends is related to quality of life (Beech and Murray 2013). One study of life satisfaction in older people with reduced ADL capacity across six European countries suggests that personal rather than environmental factors are important for life satisfaction (Borg et al. 2008). This is supported by other research which suggests that positive social relations are a significant source of satisfaction for older people (Gallagher 2012).

Belonging (social attachment to place or social insideness) has been associated with inclusive local social relations for older people (Burholt 2006, 2012; Burholt and Naylor 2005). Philip and Shucksmith (2003) have argued that a lack of belonging is associated with the absence or perceived deficiencies in family and friend networks alongside failures in other systems through which resources are allocated (i.e. private market processes, state systems and third sector).

Exclusion from social relations (or social isolation) has been associated with greater levels of loneliness in the older population. There is an extensive body of evidence on this topic, which is too vast to capture in this article. For summaries, see for example De Jong Gierveld et al. (2018) and Victor et al. (2005, 2008).

#### Social opportunities

The ERG noted that exclusion from social relations can lead to reduced social opportunities for older people such as employment, volunteering or other forms of social participation (Fig. 2). This is an example of the potential influence of the microsystem on the mesosystem (Fig. 1) and interaction with other domains of exclusion (e.g. cultural and civic participation).

Research suggests that exclusion from social relations (especially generative social relations) earlier in the life course decreases social opportunities for volunteering in later life (de Espanés et al. 2015; Urrutia et al. 2016). Similarly, research has demonstrated how important social relations throughout the life course are in securing employment in later life (Arber 2004; Phillipson et al. 2004). The evidence of the impact of exclusion from social relations on social opportunities for involvement in leisure and cultural activities is scarce. However, one study found that friends and partners played a role in stimulating participation for older people (Toepoel 2013).

#### Social cohesion

Social cohesion refers to the community and is located in the exosystem of the ecological framework (Fig. 1). The ERG noted that social cohesion is the product of complex relationships between elements that contribute to the risk for and outcomes of exclusion from social relations and is related to other domains of exclusion for older people (including cultural and civic participation) (Fig. 2).

Some academic literature focuses on the politics of belonging in terms of area disadvantage, citizenship, immigration and multiculturalism and has associated this with community social cohesion (e.g. Hewstone et al. 2005; Laurence and Heath 2008; Yuval-Davis 2006). However, Crowley (1999) argues that social cohesion is not confined to the legitimization of belonging and notions of citizenship, but is about how people perceive their location in the social world and is therefore influenced by experiences of social exclusion, including exclusion from social relations—connections to and interaction with others in the neighbourhood.

#### Health and functioning

As noted above, physical and cognitive health are included as part of the microsystem in the ecological framework (Fig. 1) as biological and neurological risks for exclusion from social relations (Fig. 2). However, the ERG also conceived health and functioning as an outcome of the interactions between different levels of the human ecological framework.

There is evidence that good social relations can help older people to maintain physical and psychological health and functioning (Courtin and Knapp 2017). There are two theoretical perspectives (buffering and direct effects) that describe the association between social relations and health and functioning outcomes (Holt-Lunstad et al. 2010). In the buffering hypothesis, social relations decrease biological stress responses to negative life events and ultimately protect older individuals from poor health (Catell 2004). For example, Cacioppo and Hawkley (2009) note that perceived social isolation contributes to poor cognitive performance and cognitive decline. On the other hand, the direct effects model suggest that social relationships shape functional healthy behaviours or promote psychological health by increasing self-esteem and purpose in life (Cohen 2004).

There is evidence to suggest that a socially integrated lifestyle (the converse of exclusion from social relations) protects against dementia and Alzheimer’s disease in the older population (e.g. Bennett et al. 2006). A meta-analysis found that less social interaction (rather than size or satisfaction with social network) increased the risk of dementia (Kuiper et al. 2015).

### The passage of time, interrelationships and feedback loops

The ERG’s conceptual model is dynamic and incorporates the chronosystem from the human ecological framework (Fig. 1), which takes into account changes in elements of the model over time (Fig. 2). For example, exclusion from social relations (objectively greater levels of social isolation or subjective assessments of quality of relationships) may differ across the life course.

In general, research has found that depsite a decline in social activities in later life, relations with family and friends remain stable (Cavalli et al. 2007). One study showed that patterns of social engagement in old age were established at least 20 years earlier (Dahlberg et al. 2018), and another study demonstrated that over the life-course network growth is greater than network shrinkage (Cornwell et al. 2014). A study by Shaw et al. (2007) found that social relationships in later life were dynamic with age-related changes varying across different dimensions (e.g. emotional, information and tangible support). Furthermore, they found significant interpersonal differences in trajectories of social relations. Similarly, research on the quality of social relations has found that this differs across the life course with some indication that relationships get better with age (Luong et al. 2010).

It has been argued that the amount of time individuals spend in a process or context generally increases the magnitude of influence on individual outcomes (Bronfenbrenner 2005). One study has suggested that prolonged social isolation may affect health and functioning through biological processes associated with the development of cardiovascular disease (Shankar et al. 2011).

The conceptual model also takes into account that factors at different levels of the model have implications for the outcomes of exclusion from social relations for older people, and these factors frequently influence one another. For example, the evidence reviewed indicated that a clinical feature of dementia is a decline in social cognition including recognition of emotions (American Psychiatric Association 2013) and insight which in turn may contribute to a decline in social relations. However, the observed decline in social functioning is unlikely to be due to brain injury alone, but is also influenced by social dynamics such as cultural values, environmental accessibility and discrimination, psychological resources and socio-emotional processes such as sense of control or resilience (Burholt et al. 2016c). Conversely, other reviews of research studies have demonstrated that loneliness (an outcome of exclusion from social relations) can lead to impaired cognitive function and decline over time (Cacioppo and Hawkley 2009; Hawkley and Cacioppo 2010). This bidirectional or reciprocal relationship suggests that social relations, cognitive impairment and loneliness reinforce each other and may be contributed to a spiral of decline (Bosma et al. 2002) (Fig. 2).

It has also been suggested that social relations are simultaneously a cause and effect of social opportunities (such as leisure or cultural participation), whereby social relations can either contributing to a virtuous circle, or a spiral of decline. For example, having good social relations increases social opportunities and can stimulate an older person to engage in certain activities, while simultaneously grasping these social opportunities can result in a greater level of social relations (Toepoel 2013).

In addition to feedback loops between social relations and outcomes, there may be collinearity between distal outcomes that may reinforce exclusion. For example, research has found an interrelationship between belonging and loneliness in later life (Beech and Murray 2013; De Jong Gierveld et al. 2015).

## Discussion

The critical review method adopted by ERG has helped to piece together research evidence from separate disciplines to identify the risks for and the outcomes of exclusion from social relations for older people. Many of the articles reviewed dealt with a distinct part of the available data which has resulted in a fragmented understanding of the phenomenon. The human ecology framework has guided our conceptualization of the interrelatedness of systems and the relationship between exclusion from social relations, risks and outcomes. The conceptual model includes risks that influence objective and subjective assessments of social relations. The subjective and objective ratings of social relations are interrelated and are influenced by psychological resources and socio-emotional processes which mediate or moderate their influence on distal outcomes such as individual well-being (e.g. quality of life, life satisfaction, loneliness and belonging), health and functioning, social opportunities and social cohesion. Sociocultural, social-structural, environmental and policy contexts also influence exclusion from social relations and distal outcomes. Finally, the model has taken into account the dynamic and interrelationships between elements of the model. We have shown that explanations for the associations between exclusion from social relations, risks and distal outcomes can be studied at different levels, ranging from biology and neurology to social-structural forces such as social policy influences. The conceptual model is a subjective interpretation of the literature and is intended to be the starting point for further conceptual development and testing.

In developing the conceptual model, we discovered deficiencies in theorizing and modelling the complexity of exclusion from social relations for older people. In this discussion, we use the conceptual model and review of the evidence to identify areas of research that are under-developed and potential pathways to or from exclusion from social relations that need to be further explored or tested.

We found that there is a fairly well-developed body of evidence about risks for exclusion from social relations for older people with regard to personal attributes, retirement, socio-economic status and exclusion from material resources and migration. On the other hand, there is less evidence concerning biological and neurological risks for exclusion from social relations. This may be because social exclusion is a sociological construct that does not feature largely in the work of these disciplines.

Historically social scientists have focused on environmental and psychosocial-structural influences on human variation, while biologists have concentrated on inherited genetic traits or evolutionary history. However, future research could benefit from an interdisciplinary approach as biology, genes and ecopsychosocial factors have a complex dynamic that influences health, behavioural and social outcomes (Belsky and Israel 2014; Goossens et al. 2015). For example, with regard to the potential impact of exclusion from social resources on specific diseases, polygenic scores can be used to study the outcomes of exposure, that is, whether exclusion mitigates or amplifies genetic risks. The construction of polygenic risk score for various physical [e.g. diabetes (Allen et al. 2010)] and cognitive diseases [e.g. Alzheimer’s disease (Escott-Price et al. 2016), Parkinson’s disease (Escott-Price et al. 2015)] and the availability of GWAS cohorts with individual ‘social’ information provide opportunities to investigate exclusion from social relations as a contour of disease risk within the older population. Furthermore, there is potential for psychophysics [defined as ‘the analysis of perceptual processes by studying the effect on a subject’s experience or behaviour of systematically varying the properties of a stimulus along one or more physical dimensions’ (Bruce et al. 2003)] to consider the implications of social cognition in terms of exclusion from social relations for older people. Successful social functioning requires an individual to be able to communicate with others, especially with respect to non-verbal communication, such as the ability to decode facial emotional expressions (Bediou et al. 2009), attribute mental states to oneself and others or to engage in mutual eye contact and joint social attention (Pfeiffer et al. 2013). These abilities (social cognition) have a biological basis in complex neural circuitry. To date, gene–environment (G × E) interaction analysis has generally been conducted with younger populations (Boomsma et al. 2005), and studies of the psychophysics of social functioning has been confined to lab-based experiments (Read 2015). Both offer opportunities for social scientist to collaborate to generate a better understanding of the biological and neurological risks for exclusion from social relations for older people.

The conceptual model developed by the ERG is novel in that it situates an evaluation of social relations (whether desires or needs are met) as central to the experience of exclusion and an essential component in the pathways to distal outcomes. While there is a substantial body of work that uses a subjective assessment approach to describe how exclusion from social relations for older people can lead to loneliness (Perlman and Peplau 1981), it has not been as rigorously tested in relation to other distal outcomes such as quality of life, life satisfaction, belonging, social cohesion and functioning. In this respect, future research should be mindful of the differences between social isolation and satisfaction with social relations, taking into account that these are not interchangeable concepts, nor necessarily stable over time and can be influenced by risks, psychological resources and processes or particular sociocultural, social-structural and environment contexts.

In the conceptual model, we posit that the evaluation of social relations by older people and any discrepancy arising between desired and achieved social relations is moderated by psychological resources and socio-emotional processes. Psychological processes (such as attribution, SOC, resilience and coping strategies) have the potential to influence the experience of exclusion from social relations. Therefore, it is essential to understand the circumstances under which older people are able to manage losses and experience good outcomes. Despite a corpus of research on cognitive processes including automatic comparison (Mussweiler et al. 2004), unrealistic optimistic comparison (Bortolotti and Antrobus 2015) and resilience (Aburn et al. 2016), theoretical and measurement models have not considered the moderating or mediating effect of psychological processes on the pathway between exclusion from social relations and outcomes for older people (for exceptions see, Burholt and Scharf 2014; Burholt et al. 2016c), and further research is required in this area.

The ERG interpretation of the evidence suggests that all elements of the conceptual model are influenced by sociocultural, social-structural and environmental contexts. However, more research is required to explore or test out some of these assumptions and to develop a deeper understanding of the relationships between these contexts. In particular, we know very little about the role of discrimination and neighbourhood safety in exclusion from social relations and distal outcomes. We also need to consider cultural effects on exclusion from social relations for older people. For example, how normative expectations about sources of support and family forms influence the extent to which social relations can protect or buffer an older person from adverse outcomes, or how the transgression of cultural values about social relations can create stigma and undermine the ‘moral status’ of an individual (Liu et al. 2008).

In the conceptual model, the *process of exclusion* from social relations for older people results in outcomes relating to individual well-being, social opportunities, social cohesion, health and functioning. As noted above, the largest body of research relates to loneliness as an outcome. Other distal outcomes of exclusion from social relations have not been so rigorously tested.

In addition to acknowledging the complexity of routes to exclusion from social relations and distal outcomes, we need to understand the dynamic interrelations between the phenomena within the human ecological framework. There have been substantial advances in statistical methods: the effect of multiple mediators and moderators can be estimated in the same model whilst controlling for other important characteristics (Hayes 2018; Muller et al. 2005). This means that large datasets can be used to test some of the complex pathways that have been conceptualized regarding associations between individual, social and environmental factors. However, a fundamental limitation of regression-based models is the inability to take into account the dynamic and reciprocal relations between factors, and discontinuities or changes over time that have been incorporated into the conceptual model of exclusion from social relations.

In other academic fields (e.g. epidemiology), academics have recognized the challenge of adopting complex system dynamic modelling to meet the demand of capturing life-course circumstances and complex interactions over time (Marshall and Galea 2015). The conceptual model (Fig. 2) has articulated a set of relationships that suggests that gerontologists should also adopt some more sophisticated modelling methods.

### Strengths and weaknesses

The critical review method typically has several weaknesses. For example, locating evidence is not as systematically rigorous as other approaches to synthesizing research, such as systematic reviews or meta-analyses, nor is there a formal requirement to describe quality assessments of material that is presented. This is because the emphasis is on identifying the most significant items in the field, the contribution of each piece of evidence to the overall conceptual model and theory development. Implicit in the deductive approach is the tendency to verify the conceptual model and to overlook opportunities to expand or refute theoretical tenets (Hsieh and Shannon 2005). Thus, critical reviews are often perceived as too subjective in the way that analyses are conducted and conclusions drawn. However, we believe that using a group approach has meant that we have held our biases in abeyance. Through a critical review of the evidence by email and in face-to-face meetings, validity claims have not been accepted or rejected with discursive consideration. Jointly, we have determined the justifiability of the conceptual model. Nevertheless, it is important to highlight that this critical review has aggregated the data in such a way that the interpretation and conceptual model are necessarily subjective and other alternative models and/or additional domains within the model may exist. For example, there may be alternative psychological resources that influence social exclusion but were not included in the review of the literature.

## Conclusion

The critical review and conceptual model of exclusion from social relations for older people provide a starting point for further phases of conceptual development and systematic evaluation(s). Many of the potential relationships between levels and elements of the model require further investigation, and in order to address this gap, interdisciplinary research is required. Furthermore, sophisticated analytical tools and an interdisciplinary approach are required to understand the underlying biological and ecopsychosocial associations that contribute to individual and dynamic differences in the experience of exclusion from social relations for older people.
